# Steep and deep: Terrain and climate factors explain brown bear (*Ursus arctos*) alpine den site selection to guide heli-skiing management

**DOI:** 10.1371/journal.pone.0238711

**Published:** 2020-09-23

**Authors:** Anthony P. Crupi, David P. Gregovich, Kevin S. White

**Affiliations:** Division of Wildlife Conservation, Alaska Department of Fish and Game, Douglas, Alaska, United States of America; Bowling Green State University, UNITED STATES

## Abstract

Winter recreation and tourism continue to expand worldwide, and where these activities overlap with valuable wildlife habitat, there is greater potential for conservation concerns. Wildlife populations can be particularly vulnerable to disturbance in alpine habitats as helicopters and snowmachines are increasingly used to access remote backcountry terrain. Brown bears (*Ursus arctos*) have adapted hibernation strategies to survive this period when resources and energy reserves are limited, and disturbance could negatively impact fitness and survival. To help identify areas of potential conflict between helicopter skiing and denning brown bears in Alaska, we developed a model to predict alpine denning habitat and an associated data-based framework for mitigating disturbance activities. Following den emergence in spring, we conducted three annual aerial surveys (2015–2017) and used locations from three GPS-collared bears (2008–2014) to identify 89 brown bear dens above the forest line. We evaluated brown bear den site selection of land cover, terrain, and climate factors using resource selection function (RSF) models. Our top model supported the hypothesis that bears selected dens based on terrain and climate factors that maximized thermal efficiency. Brown bears selected den sites characterized by steep slopes at moderate elevations in smooth, well-drained topographies that promoted vegetation and deep snow. We used the RSF model to map relative probability of den selection and found 85% of dens occurred within terrain predicted as prime denning habitat. Brown bear exposure to helicopter disturbance was evident as moderate to high intensities of helicopter flight tracking data overlapped prime denning habitat, and we quantified where the risk of these impact was greatest. We also documented evidence of late season den abandonment due to disturbance from helicopter skiing. The results from this study provide valuable insights into bear denning habitat requirements in subalpine and alpine landscapes. Our quantitative framework can be used to support conservation planning for winter recreation industries operating in habitats occupied by denning brown bears.

## Introduction

Winter recreation and tourism activities have steadily increased worldwide over the past several decades [1]. Iconic ski films such as Warren Miller’s ‘Steep and Deep’, gave rise to an era of extreme skiing and inspired a ubiquitous quest for untracked, big mountain powder. The growth in the winter tourism industry can generate huge economic benefits to mountain communities [1], though potentially at considerable costs to wildlife species occupying areas used by recreationalists [2]. Expansion of winter recreation activities into alpine habitats causes conservation concerns for animals experiencing human disturbances at a time of limited resources and unfavorable climatic conditions [3–5]. Conservation of species that use mountain habitats during winter relies on sound science to guide management decisions.

Weather conditions in winter can be severe and numerous mammal species have adapted energy minimizing strategies, such as hibernation, to survive this period when food resources and energy reserves are limited [6]. Overwinter survival and successful reproduction of brown bears (*Ursus arctos*) depend on dens that provide a stable, dry, and insulated environment for up to six months, making the hibernacula they choose a fundamental aspect of their life cycle. The physiology of hibernation allows bears to conserve energy by reducing activity level, body temperature, and metabolism [7, 8]. Female reproductive success is positively related to body mass and fat content [9], and female bears with dependent offspring expend more energy than other cohorts during hibernation due to the costs associated with gestation and lactation [10, 11]. Lactating females entering the den with more stored fat energy produce larger cubs with higher survival, especially when allowed to nurse their cubs longer in the den [12].

Disturbance to brown bears caused by winter recreation, aircraft activity, development, and resource extraction can negatively influence brown bear populations [13–18]. Individual behavioral and physiologic responses to disturbance may result in population level concerns when impacts affect survival and reproduction [19, 20]. During hibernation brown bears can be easily disturbed [21], which contributes to increased mass loss [22], increased stress, long-term displacement from favored habitats, den abandonment, and bear mortality [21, 23]. The benefits of hibernation may be eclipsed by winter disturbances that reduce an individual’s energetic reserves, reproductive potential, and survival. Despite the critical importance of a den to a bear’s survival, extensive human activities can influence habitat use and result in avoidance of suitable denning habitat [17, 24].

Exposure to aircraft disturbance has been shown to elicit a strong stress response in bears [25, 26]. Low-level flights can potentially disrupt normal bear behaviors including hibernation, denning duration, and springtime foraging activity [27]. In most remote denning areas human activity is limited by access. However, helicopters enable access to remote and isolated geographies that otherwise experience limited human activity. Compared to other causes of disturbance, high decibel (dB) helicopter sound (approx. 100 dB) influences large tracts of habitat with animals overtly responding from up to several kilometers away [28, 29]. Disturbance severity increases with duration, frequency, and intensity which cumulatively contribute to large physiologic and energetic costs to the animal [30]. Infrequent helicopter overflights with no landings at altitudes greater than 500 m are generally believed to have minimal effect on bears. However, extensive helicopter operations below 500 m, with or without landings, are likely to adversely affect brown bears [30].

In Alaska and Canada, the helicopter-skiing (hereafter, heli-skiing) industry has expanded over the past two decades, accessing backcountry terrain during the critical late-winter period from February to May [31, 32]. The number of heli-skiing permits and authorized heli-skiing terrain has more than doubled in some areas [33], due to increased demand and economic pressure to reduce regulations. Additional activity in alpine environments poses a potential risk to species susceptible to disturbance. Winter habitat use by bears [34] and other species, such as mountain goats (*Oreamnos americanus*) [35] and wolverines (*Gulo gulo*) [36], has been negatively associated with heli-skiing areas. In addition to wildlife disturbance and habitat avoidance, heli-skiing and snowmobile use, can trigger avalanches on steep slopes (>25°) [37], which increases the risk of fatality for both people [38, 39] and bears [40, 41]. Whether the consequences of these risks are acceptable is a management decision reliant upon empirical data concerning wildlife habitat requirements necessary to inform public opinion.

To obtain information about the landscape features selected by brown bears for denning, and thus provide a means for data-based spatial mitigation of disturbance, we flew aerial surveys in spring to locate dens in alpine and subalpine habitats. Monitoring species distribution in winter alpine environments, where traditional mark-resight approaches can be logistically challenging and economically unviable, poses a unique challenge [42–44]. Flying aerial surveys to identify dens is an established technique that has been used in other studies for more than 50 years, affording wildlife biologists a cost-effective method to obtain information on brown bear denning ecology in open habitats [45–49]. Using slow-flying, maneuverable aircraft with good visibility, biologists have learned about den habitat requirements by searching mountainous terrain above tree-line looking for den entrances highlighted by dark mounds of excavated earth and stained tracks on a white snow background. In addition to aerial observation, dens are also commonly located from bears instrumented with radiocollars [17, 50, 51] and then assessed in relation to a variety of landscape characteristics associated with denning habitat [45, 52, 53].

Our objective was to characterize brown bear den site selection and develop a predictive model of brown bear denning habitat in non-forested, alpine and subalpine landscapes, habitat most commonly used by heli-skiing. We developed a quantitatively rigorous, data-based approach to aid decision-making associated with regulating heli-skiing with the intended benefit of minimizing disturbance to and promoting viability of bears denning in alpine habitats. We tested a suite of ecological hypotheses to determine the factors associated with den site selection in open habitats. At the most fundamental level, we predicted that bears would select den site locations based on topography alone (i.e., because bear dens require certain basic structural attributes). Using this terrain-only model, we predicted that bears would select mid-range slopes, moderate elevations, and topographic position with greater relief to provide security. Bears, especially females, benefit from lengthy (5–6 months) denning periods, and we suspected bears conserved energy by denning in locations that produced deep and stable snow conditions. We built upon the terrain model by including climate related factors to test the hypothesis that bears select dens to maximize thermal efficiency. We predicted that bears would select dens that facilitate accumulation and persistence of deep snow for insulation, provide vegetation for stability, inhibit solar radiation to limit early snow melt, and occur in well-drained topographies that prevent den flooding. We also considered several more complex den selection models to explore potential interactive effects between terrain factors and climate covariates. Finally, to assist managers tasked with mitigating potential disturbance to denning brown bears, we developed a risk assessment framework that involved spatially overlapping brown bear denning habitat (based on RSF models) with heli-skiing intensity (from heli-skiing flight tracks) to identify areas of concern for potential impact risks in alpine and subalpine den habitat.

## Materials and methods

### Study area

**Fig 1:**
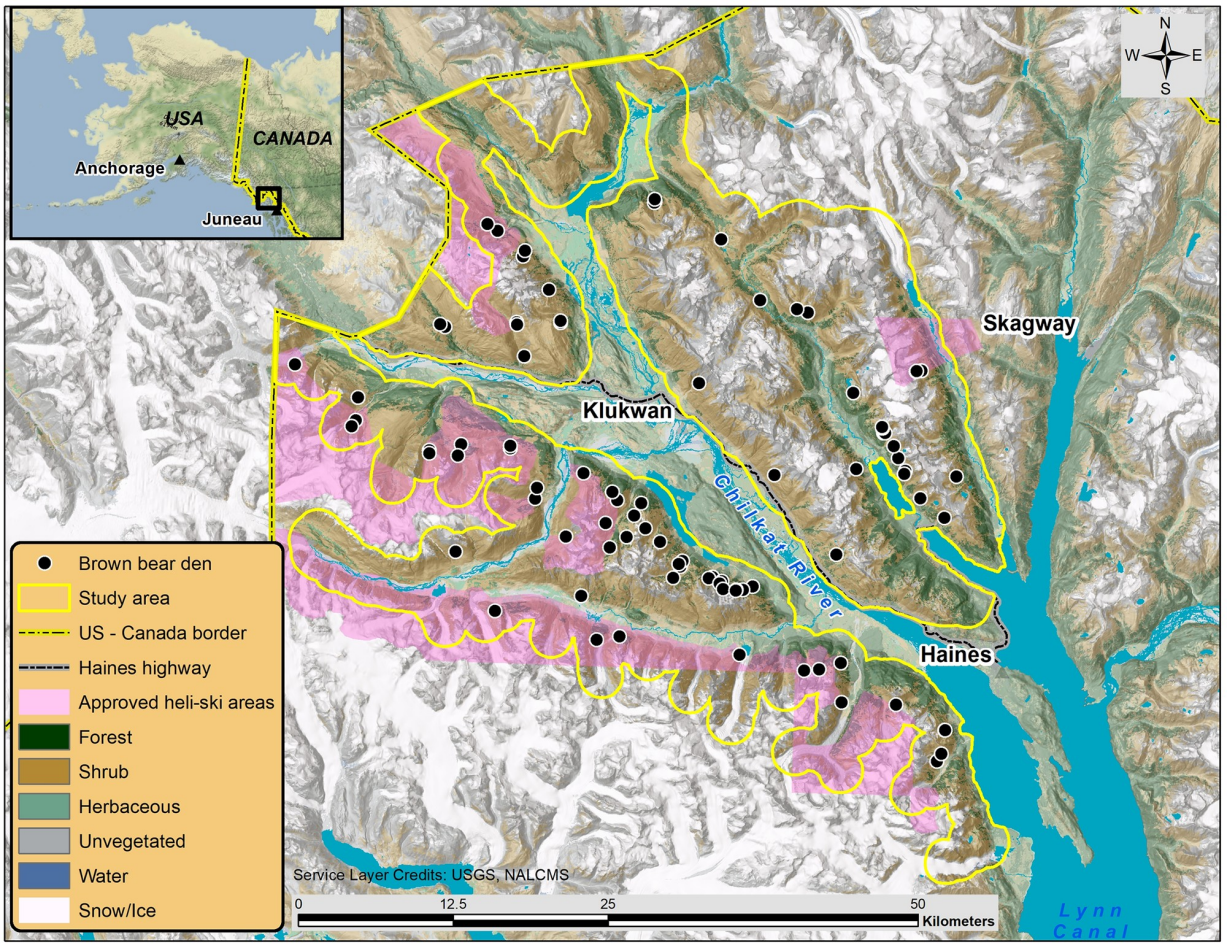
Study area used to describe brown bear alpine den site selection in Southeast Alaska, U.S.A., 2008–2017. Generalized brown bear den locations are denoted by black circles, study area is delineated by the solid yellow line, approved heli-skiing areas are shaded violet, and dominant land cover classes are detailed, republished from [57] under a CC BY license, with permission from Commission for Environmental Cooperation, original copyright 2020.

The mainland coast of northern Southeast Alaska (SEAK) near the town of Haines (59°17′ N, 135°56′ W) is a mountainous region bordered by the Pacific marine environment to the south and the dry interior climate of British Columbia and Yukon Territories, Canada to the north. The region has a moist maritime climate and the majority of snowfall accumulates between November and April (Haines Border Station elevation 275 m, mean annual snowfall 6.3 m) [54]. We designed our study area to broadly extend to where heli-skiing activities occur or have been proposed and include only areas that were potentially visible during the aerial surveys (i.e., open habitats near or above tree-line). We delineated the 1,052 km^2^ study area by buffering the aerial survey route by 1,500 m and excluding forested habitat, elevations below 300 m, and glaciers, as brown bears rarely occupy glacial habitat [55, 56] (Fig 1). The glacially influenced terrain is complex and variable throughout the study area with elevation ranging from 300–2,048 m. The remote, rugged terrain typically limits human access in winter. However, since 2000, a helicopter-supported ski industry has provided backcountry access.

The majority of study area lands are administered by the Alaska Department of Natural Resources and the Bureau of Land Management (BLM). At the request of the local community, the Haines Borough took an active role in managing the heli-skiing industry on state lands through a tour permitting process. The BLM manages heli-skiing on its lands through a separate permitting procedure [58]. Combined, the approved heli-skiing area totals 957 km^2^ in five separate units. The Haines Highway, along the Chilkat River, is plowed during winter and provides limited road access to the study area. There are also a few unmaintained forest roads that serve as winter trails for snow machines and snowcats to access alpine ski terrain.

The alpine and subalpine habitats surveyed were a heterogeneous mix of herbaceous meadow, unvegetated scree slope, and an alder (*Alnus spp*.) and willow (*Salix spp*.*)* shrub community, with composition similar to a nearby study area described by Boggs et al. [59]. The lower limit of the survey area transitions into subalpine forest which is dominated by mountain hemlock (*Tsuga mertensiana*), western hemlock (*Tsuga heterophylla*), and Sitka spruce (*Picea sitchensis*), some exhibiting krummholz vegetation. The formation of coniferous forest is influenced by several factors including elevation, slope, persistence of snow and ice, avalanche frequency, and other environmental conditions. Within the study area we digitized the forest line (the upper limit of continuous tree canopy) [60] from high-resolution imagery (SPOT-5) and land cover data [61], and found the forest line ranged from 300–900 m, with a mean elevation of 480 ± 168 m.

Wildlife populations in the study area are of great value to the local community and economy [62]. Hunters primarily harvest moose (*Alces alces*), black bears (*Ursus americanus*), and mountain goats for subsistence, as well as brown bears and wolves (*Canis lupus*). An expanding cruiseship industry supports economic growth in wildlife viewing and adventure tourism. Coastal mainland regions, such as our study area, typically support moderate brown bear densities (50–150 bears/1,000 km^2^) due to the seasonal availability of salmon [63].

### Aerial survey

To locate and characterize dens in alpine habitats we conducted aerial surveys during the period of den emergence and prior to deciduous vegetation leaf-out. Surveys were flown in late-April (2015–2017) during daylight hours (9:00–18:00 AKDT) at speeds of 100–120 km/hr depending on wind speed and direction. Our survey protocol involved flying a continuous transect [64] above the forest line and below hanging glaciers where visibility is suitable, terrain is typically open, and bear dens can be detected. A pilot and single observer conducted the surveys from a fixed-wing Piper Supercub (PA-18) typically flying at 150–300 m above ground level, scanning habitat with the use of binoculars for evidence of bear dens and tracks. Survey altitude was adjusted throughout the flight to accommodate changes in the forest line, snow line, and other terrain obstructions, allowing us to survey the range of elevations occurring within the study area. This protocol enabled us to readily scan habitat within a 1,500 m buffer of the transect, encompassing most suitable alpine denning habitat within our survey footprint. Previous aerial survey work using line transect sampling in Alaska showed that bears were detected within 1,500 m of the aircraft with maximum detectability near 600 m [64], though we consider those detectability functions to represent minimum distances because detections under our high-contrast conditions were more favorable. While in practice it was possible for us to detect dens from the aircraft at distances greater than 1,500 m, because the extensive coverage of mud stained snow, we felt it was only within this distance threshold that we could consistently detect dens without risk of bias. We believe this approach used to define the study area was conservative, relative to our actual survey capabilities.

When a den site was located the pilot circled the den site while the observer photographed the den and surrounding area with a digital single-lens reflex (DSLR) camera and recorded the latitude and longitude coordinates on a handheld GPS. In addition to the dens identified during aerial surveys, we supplemented our den site data with data collected from three GPS-collared female brown bears that denned in the study area between 2008–2014. The GPS collars collected daily locations, temperature, and activity sensor data during hibernation. To verify remotely-sensed terrain and habitat determinations, we recorded habitat information surrounding the den sites, including slope of the terrain (steep, moderate, flat), life zone (alpine, subalpine), and vegetation type (herbaceous, shrub, subalpine forest, unvegetated, and snow). Most bear tracks were observed near the den site, but some tracks were left by bears traversing the alpine. We mapped features along the survey route using ArcGIS (ESRI 2014, ver. 10.3). Photographs of den sites, bear tracks, ski runs, and snow conditions were geocoded with positional data using RoboGeo (Pretek, Inc, ver. 6.3.2) and evaluated to confirm den sites, vegetation type, distribution of bear and skiing activity, and snow level.

Specific bear den locations are confidential under Alaska state law (AS 16.05.815(d)), hence we depict generalized locations on mapping outputs. Capture protocols were approved by the Institutional Animal Care and Use Committee (08–12) following strict guidelines [65].

### Den site selection factors

Based on previous research findings, we predicted that brown bear den site selection would vary with slope [34], elevation [66], habitat type [67], subalpine forest structure [68], and snow depth [69]. We developed fine-scale habitat factors (Table 1) from a remotely sensed Interferometric Synthetic Aperture Radar (IfSAR) digital elevation model [70] with 5-m resolution using ArcGIS Spatial Analyst, R [71], GRASS [72], and SAGA GIS [73]. Using a land cover classification (30-m resolution) for SEAK [61], we aggregated dominant classes into five cover type covariates and calculated their frequency for elevations above 300 m; forest (26%), shrub (24%), herbaceous (10%), unvegetated (25%), and snow/ice (15%). Based on preliminary model selection results, bears did not select den sites associated with land cover type and so it was removed from further consideration. As den sites within the forest were not visible from the air, we developed a vegetation height index (VHI) to mask forested habitats (>5 m) from the study area. VHI was calculated as the difference between the IfSAR Digital Terrain and Digital Surface Models (DTM and DSM), which estimate the elevation of the bare earth and canopy height, respectively. We then evaluated if bear den site selection was explained by low vegetation cover (<5 m, e.g., shrub or herbaceous) as we expected the presence of vegetation and its roots would provide structure to the soil and promote den stability. We extracted covariate values for each den site and available location (see below for description of available habitat) to test hypotheses related to brown bear den site selection (Table 2).

**Table 1:** Terrain, climate, and land cover habitat factors, descriptions, abbreviations (code), and data source used to predict brown bear den site selection in Haines, Alaska, U.S.A., 2008–2017.

Category	Factor	Description	Code	Data source
Terrain	Elevation	Elevation (m)	dtm	IfSAR-DEM; ArcGIS Spatial Analyst
	Slope	Slope (degrees)	slope	IfSAR-DEM; ArcGIS Spatial Analyst
	Topographic position index	Topographic position index based on the slope of surrounding cells	tpi	IfSAR-DEM; SAGA GIS tool [73, 75]
	Vector ruggedness measure	Vector ruggedness measure captures complexity in smooth vs. irregular terrain	vrm	IfSAR-DEM; Python script derived from [76]
	Vegetation height index	Canopy height calculated as difference between vegetation height and earth surface	vhi	IfSAR-DEM
Climate	Snow load index	Snow load index, product of standardized aspect for prevailing wind (135°) and elevation	snow.load	IfSAR-DEM; script derived from [66]
	Solar radiation	Solar radiation calculated for 1 April	solrad	IfSAR-DEM; GRASS GIS r.sun function
	Topographic wetness index	Topographic wetness index based on slope and drainage from upstream catchment area- twi	twi	IfSAR-DEM; SAGA GIS tool [73, 77]
Land Cover	Forest	Forest characterized by conifers or deciduous trees		Land cover classification [61]
	Herbaceous	Herbaceous forbs and graminoids—used as the reference class		Land cover classification [61]
	Shrub	Land cover types dominated by deciduous shrubs		Land cover classification [61]
	Unvegetated	Unvegetated habitats including bare ground, non-vascular, and water		Land cover classification [61]
	Snow/Ice	Snow and ice		Land cover classification [61]

**Table 2:** A priori candidate models used to predict brown bear den site habitat selection in the alpine near Haines, Alaska, U.S.A., 2008–2017.

Model	Hypothesis- brown bear den site selection	Model factors	Prediction
1&2	Terrain–position on landscape drives selection	dtm, dtm_sq, slope, slope_sq, tpi, (tpi_sq)	select topography based on moderate elevations, with mid-ranged slopes, and greater topographic relief
3&4	Terrain and vegetation–selection is a combination of landscape position and vegetation height	dtm, dtm_sq, slope, slope_sq, vhi, (vhi_sq), tpi, (tpi_sq)	select terrain with moderate elevations, mid-range slopes and shrub vegetation
5&6	Terrain complexity–landscape terrain with complex texture is selected for denning	vrm, (vrm_sq), tpi, (tpi_sq)	select highly rugged landscapes with high topographic relief for security
7&8	Climate–dry snow–select habitat with deep snow deposition and avoid wet terrain	snow.load, (snow.load_sq), twi, (twi_sq)	select habitat that promotes deep snow in well-drained topographies
9&10	Climate–thermal insulation–choose sites with deep snow and low solar to facilitate long den occupation	snow.load, (snow.load_sq), solrad, (solrad_sq)	select for thermal insulation in areas with high snow deposition
11&12	Terrain and climate–slope+vegetation+thermal–select habitats with a combination of terrain, vegetation, and thermal insulation climate features, irrespective of elevation and ruggedness	slope, (slope_sq), snow.load, (snow.load_sq), solrad, (solrad_sq), tpi, (tpi_sq), vhi, (vhi_sq)	select based on good insulative values in shaded terrain with moderate slope, shrub vegetation, and high topographic relief, irrespective of elevation and ruggedness
13&14	Terrain and climate–terrain+vegetation+dry/ thermal snow–select terrain combinations of slope, elevation and ruggedness with vegetation heights that promote dry snow climates	dtm, dtm_sq, slope, slope_sq, snow.load, [solrad], twi, vhi, [vrm]	select combination of moderate elevation and mid-range slopes in non-rugged terrain that promotes snow deposition in well-drained topographies with shrub vegetation
15–18	Terrain and climate–complex—global model–den selection driven by the interaction between terrain, vegetation height, and ruggedness in landscapes that promote dry snow climates	dtm, (dtm_sq), slope, (slope_sq), snow.load, (snow.load_sq), [solrad], (solrad_sq), [tpi], (tpi_sq), twi, (twi_sq), vhi, (vhi_sq), vrm, (vrm_sq)	select non-rugged terrain with moderate slopes and elevations, high topographic relief with low solar energy in well-drained and vegetated topographies that promote snow deposition

(factor_sq) = models vary by the omission of quadratic terms; [factor] = models vary by the omission of the given factor.

We derived several terrain factors from the IfSAR DTM to describe topographic characteristics we predicted bears would select for denning. In addition to extracting values for elevation and slope, we calculated a topographic position index (TPI). Using a moving circular window analysis (140-m) [74], we calculated the difference between the elevation at each cell and the mean elevation of the surrounding cells using a morphometry module tool within the Terrain Analysis toolset in SAGA GIS [75]. We used TPI to describe the position of a den site relative to the surrounding landscape (e.g., ridge top, middle slope, valley bottom) with positive values indicating convex terrain features and negative values reflecting concave topography. To examine the importance of complexity between smooth and irregular terrain we calculated a vector ruggedness measure (VRM) using a 15-m neighborhood analysis to describe the three-dimensional texture of the terrain surface [76]. We expected this terrain feature would distinguish areas where bears were able to safely traverse and find suitable digging habitat.

We tested our prediction that bears selected den sites that promoted thermal efficiency using three climate factors that we believed would maximize denning duration. Snow cover affords excellent insulative properties due to its low thermal conductivity, and is thought to shelter bears from unfavorable weather conditions while maintaining relatively stable soil temperatures [78]. We calculated a snow load factor as the product of two elements, elevation scaled (0–1) across the entire range of available elevations, and a bearing exposure component that was at a maximum opposite from the prevalent wind bearing [66]. In the winter months, the predominant wind direction (135°) was determined by assessing wind data associated with snowfall events [54, 79]. To determine the effect of hydrological processes and soil moisture on bear den site selection, we calculated a topographic wetness index (TWI) using slopes generated from the IfSAR DTM as a function of the specific catchment area [77]. We predicted that bears would select drier, well-drained habitats to reduce potential for den flooding [48] and improve thermal insulation as snow density negatively influences thermal conductivity [80]. Solar radiation was derived via GRASS integrated in QGIS to depict the incoming solar energy varying with changes in elevation, aspect, and slope [81]. We calculated solar radiation for 1 April to represent the den conditions prior to the expected onset of den emergence. We anticipated that bears would select den sites that reduced solar insolation (solar radiation energy) to minimize snow ablation (melting and evaporation) and maintain thermal insulation properties. All continuous factors were standardized (x-x̄ /SD(x)) prior to analysis. Temperature and snowfall vary within the study area along a maritime to interior climatic gradient, therefore, we tested for terrain factor differences among different mountain ranges using ANOVA and Tukey’s HSD (Proc GLM, SAS Institute Inc., ver. 9.3).

### Habitat selection model

To measure brown bear den site selection, we developed a resource selection function (RSF) model. RSFs model relative probability of selection by statistically comparing the environmental attributes of observed den site locations to random available locations that characterize the surrounding environment. To estimate resource availability, we generated randomly distributed locations at the scale of the study area (second-order selection) [82, 83] at a mean density of 500 locations per km^2^ [84]. Habitat characteristics of den sites were contrasted with the available points following a Design II approach [85]. Each individual brown bear den observed along the survey route was included in the population-level RSF, except for 1 den that was masked from the study area (i.e., VHI > 5).

We built models using the *glm* function in R [71], to describe the relationship between animal use and habitat factors via the logistic regression equation:

$$w(x)=exp(\beta_{1}x_{1}+\beta_{2}x_{2}+\ldots +\beta_{n}x_{n})$$ (1)

where *w(x)* is proportional to the relative probability of selection for each individual den. Potential habitat factors were first screened for collinearity (|*r*| < 0.7), and variance inflation factors (VIF) for all covariates included were < 2 [86]. RSF scores were proportional to the relative predicted probability of selection of a given resource unit, hence we did not use the intercept [87]. We tested a suite of 18 biologically plausible candidate models and selected the most parsimonious model with the lowest corrected Akaike Information Criterion (AICc) [88]. We interpreted the effect of each factor, generated individual factor effect response curves with other factors held to their mean, and considered coefficients not informative if the 95% confidence interval included zero [89].

### Model validation

We validated the predictive capability of the models using *k*-fold cross-validation with five iterations [87, 90]. RSF scores were calculated for all available points, and these scores were then ordered and split into 10 equal-sized bins and ranked from low to high. The mean RSF score of each bin was divided by the sum of these means to yield the expected proportion of locations in each bin. The RSF scores of the validation data were similarly split using the same breakpoint values used to split the available points. This yielded the observed proportion of values in each bin. Larger Spearman’s rank correlation values indicate concordance and proportionality between the rankings of observed versus expected values and thus the predictive capability of the model [87, 90, 91]. This process was iterated 100 times and we reported the mean Spearman’s correlation coefficient and range.

We generated a continuous output surface map that spatially described the relative probability of den site selection using the coefficients of factors included in the top model (i.e., model with the lowest AIC_c_ score). The RSF score predictions were divided into five equal area bins by sorting the raw RSF scores from lowest to highest, and then selected breakpoints such that there were an equal number of pixel values in each bin. The following values represent the upper limit break point values for each relative probability class: low (2.83x10^-2^); low-moderate (2.18 x10^-1^); moderate (6.38 x10^-1^); moderate-high (1.45); and high (13.86). We classified prime bear denning habitat as the top two relative probability classes [92].

### Heli-skiing—Denning habitat overlap

We developed a helicopter impact risk surface depicting where the highest quality bear denning habitat was exposed to the greatest intensity of helicopter activity. First, we quantified the number of bear dens found within and outside of permitted ski areas. Then, to quantify heli-skiing intensity, we converted heli-skiing flight tracking data from the three permitted commercial operators during the 2011 ski season (Haines Borough, unpublished data) into a kernel density raster (i.e., heli-skiing intensity). We created this utilization distribution from 9,790 helicopter flight tracking locations using Geospatial Modelling Environment [93] with a least-squares cross-validation bandwidth estimator. The resulting heli-skiing intensity raster was classified into five quantile bins ranging from low to high intensity. Finally, we multiplied the heli-skiing intensity raster and the brown bear denning RSF to spatially identify areas with the highest potential for disturbance impact risk to denning bears.

## Results

### Aerial survey

**Fig 2:**
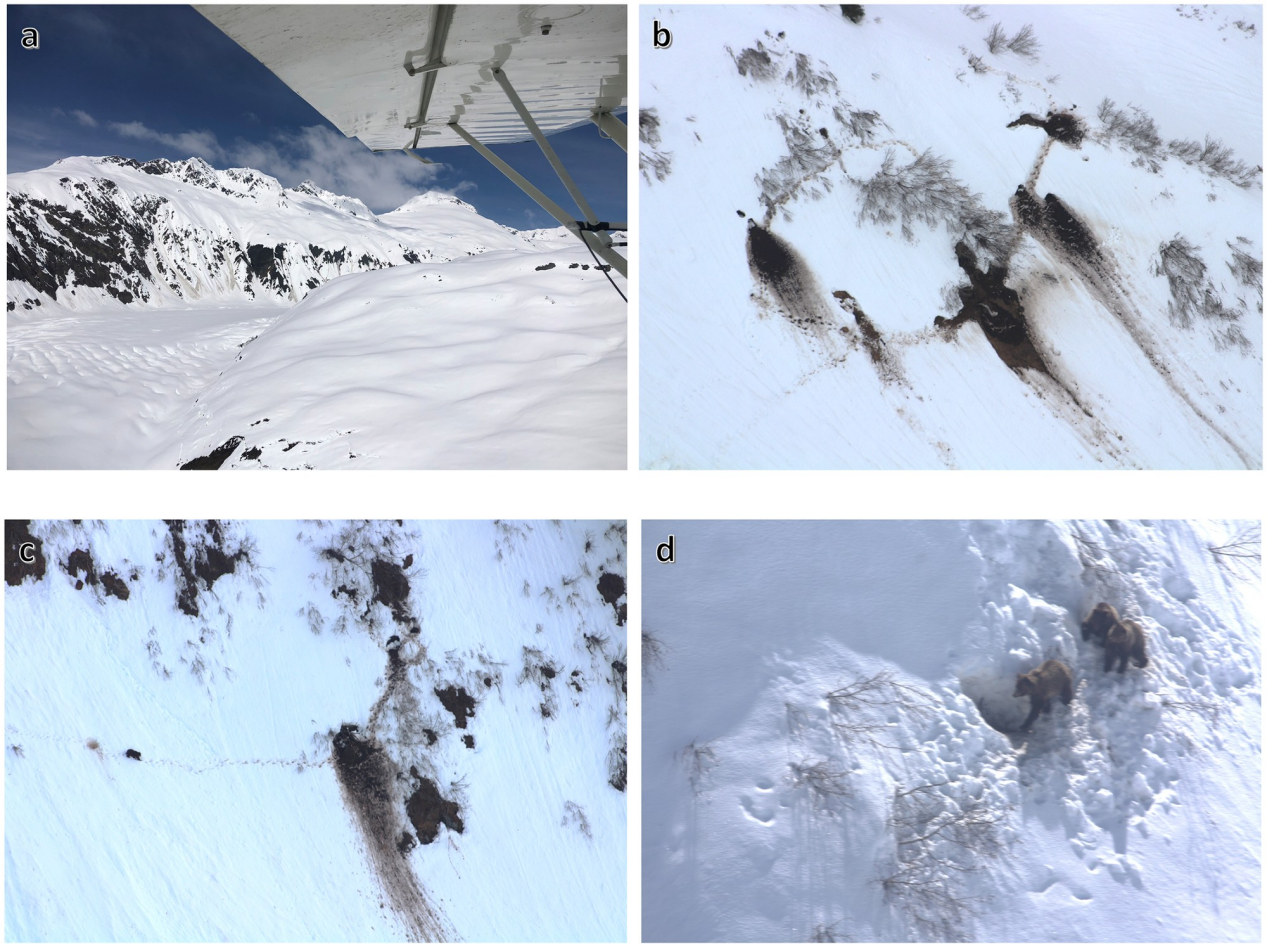
Photographs taken during aerial surveys of brown bear den site selection in alpine and subalpine habitats near Haines, Alaska, U.S.A. a) View of the study area from fixed-wing aircraft while scanning for dens, b) multiple den sites excavated in earth and surrounded by shrub habitat at 983 m elevation on 36° slope, c) bear observed near den site after emergence, d) female with two cubs observed at den entrance.

We conducted three aerial surveys during spring 2015–2017, detected 81 brown bear dens, and identified eight additional dens from three GPS-collared bears. During surveys, snow cover was generally continuous and varied in estimated depth between 1–3 m. The majority of dens (85%) were located in alpine habitat, the remainder (15%) were in subalpine habitats. Tailings piles of excavated soil near the den entrance and dirty bear tracks surrounding the den site were characteristics consistently documented at snow-covered alpine dens (Fig 2). We observed 70 alpine dens in shrub habitat, five in herbaceous alpine cover, one den was excavated in unvegetated alpine terrain, and 13 dens were surrounded by subalpine forest. On 10 occasions we observed bears in the alpine during the survey flight, eight of those bears were still at their den site, and five of the independent bears sighted were adult females with offspring.

Brown bears occupied dens across a range of available elevations and the mean den site elevation was 755.0 ± 152.7 m (Table 3). We found that den site elevation was consistent between dens identified by GPS-collared bears (mean 831.6 ± 178.2 m) and those observed by aerial survey (mean 747.9 ± 141.5 m). All GPS-collared bear dens were excavated in alpine habitat above the forest line. Bears used dens on slopes averaging 35.2° ± 10.3°, and more than 90% of the bear dens were located on slopes between 20° and 50°. Surveyed mountain ranges extend thru the climatic gradient of the study area, yet despite the geographic and climatic heterogeneity we found bears occupied dens of similar elevation (*F*_6,82_ = 1.53, *p* = 0.18) and slope *(F*_6,82_ = 1.57, *p* = 0.17) among areas (S1 Fig). Bears used 57 dens (64%) oriented on northeast to east or southwest to west facing slopes and 32 dens (36%) had north to northwest or south to southeast exposure (S2 Fig).

**Table 3:** Den site terrain and climate factor summary statistics from observed den sites and mean values of available factors used to predict brown bear den site selection in Haines, Alaska, U.S.A., 2008–2017.

Covariate	Min.	Max.	Mean	Median	SD	Mean avail
Elevation (m)	414.00	1161.00	755.05	744.50	152.67	908.15
Slope (degrees)	5.71	60.79	35.20	35.93	10.29	30.29
Snow load	0.05	1.00	0.52	0.50	0.26	0.48
Topographic wetness index	0.90	3.96	2.09	1.99	0.65	2.20
Vector ruggedness measure	0.00	0.09	0.02	0.02	0.02	0.03
Vegetation height index	-1.57	4.93	0.76	0.43	1.53	1.88

### Apparent disturbance of GPS-collared bear by heli-skiing

During three consecutive winters (2008–09 through 2010–11), a female brown bear instrumented with a GPS collar excavated a den in the same location at 1,025 m elevation, with little variation in den duration (175–190 days) or mean emergence date (5 May). Her reproductive status varied in these years from single adult to female with dependent offspring. Heli-skiing was first authorized in her denning area in February 2011 when she had two yearling cubs. Flight track data were recorded 400–1,000 m from the den site on 13 and 18 April 2011. Bear activity level recorded on the collar during the heli-skiing flight on 18 April increased when compared to the four days before and after the event (28.8 ± 57.3 vs. 12.7 ± 33.8). Immediately following this flight, the bear abandoned its den (den duration decreased to 147 days), evidenced by the temperature sensor declining to outside ambient temperatures and movement away from the den site. These bears did not establish a new den and remained active in the snow covered alpine until May. This bear was monitored with a GPS collar for three additional winters in which she did not return to this area to den and selected a den site outside of the permitted heli-skiing area.

### Habitat selection model

The top den site habitat model included a combination of specific terrain and climate factors (Table 4). The most parsimonious model supported our hypothesis that brown bears selected terrain combinations of slope, elevation, vegetation height, and ruggedness in well-drained topographies at sites that promoted snow deposition (Table 5). The model had strong predictive capability as cross-validation resulted in a mean Spearman’s correlation coefficient of 0.83 (SD: 0.04, range: 0.74–0.91). The top model was strongly selected over other competing models (80% AIC_c_ weight and > 2 Δ_i_ AIC_c_ of second model).

**Table 4:** Resource selection function model AIC_c_ scores from all models predicting brown bear denning habitat in Haines, Alaska, U.S.A., 2008–2017.

Model	Model hypothesis	K	AIC_c_	Δ_i_ AIC_c_	AIC_c_ wt
14	Terrain and climate–terrain+vegetation+dry snow	8	1589.709	0.000	0.797
17	Terrain and climate–complex model (sq)	10	1592.607	2.898	0.187
18	Global model–all factors	16	1598.023	8.314	0.012
16	Terrain and climate–complex model [vrm] (vrm sq)	14	1601.572	11.863	0.002
15	Terrain and climate–complex model [vrm] (sq)	10	1602.730	13.022	0.001
13	Terrain and climate–terr+veg+thermal [vrm] (sq)	9	1609.430	19.721	0.000
4	Terrain and vegetation (sq)	8	1619.961	30.252	0.000
2	Terrain	6	1622.063	32.354	0.000
1	Terrain (sq)	5	1626.510	36.801	0.000
3	Terrain and vegetation	6	1626.694	36.985	0.000
12	Terrain and climate–slope+vegetation+thermal	12	1672.148	82.439	0.000
11	Terrain and climate–slope+vegetation+thermal (sq)	7	1686.528	96.819	0.000
7	Climate–dry snow (sq)	2	1696.858	107.149	0.000
9	Climate–thermal insulation (sq)	2	1697.307	107.598	0.000
8	Climate–dry snow	4	1700.351	110.642	0.000
10	Climate–thermal insulation	4	1700.585	110.876	0.000
5	Terrain complexity(sq)	2	1709.334	119.625	0.000
6	Terrain complexity	4	1710.190	120.481	0.000

(sq) = model does not include quadratic terms; [factor] = models vary by the omission of the given factor.

**Table 5:** Parameter estimates (β), 95% confidence intervals (CI), and standard errors (SE) included in the top model of brown bear den site selection in Haines, Alaska, U.S.A., 2008–2017.

Model covariates	β	95% CI	SE
Elevation (m)	**-1.91**	-2.65– -1.18	0.38
Elevation^2^	**-1.78**	-2.41– -1.13	0.33
Slope (deg.)	**0.64**	0.31–0.98	0.17
Slope^2^	-0.17	-0.39–0.04	0.11
Snow load	0.23	-0.03–0.44	0.12
Topographic wetness index	-0.37	-0.75–0.02	0.20
Vector ruggedness measure	**-0.48**	-0.89– -0.08	0.21
Vegetation height index	0.18	-0.1–0.47	0.15

Bold coefficients indicate CIs not overlapping 0.

**Fig 3:**
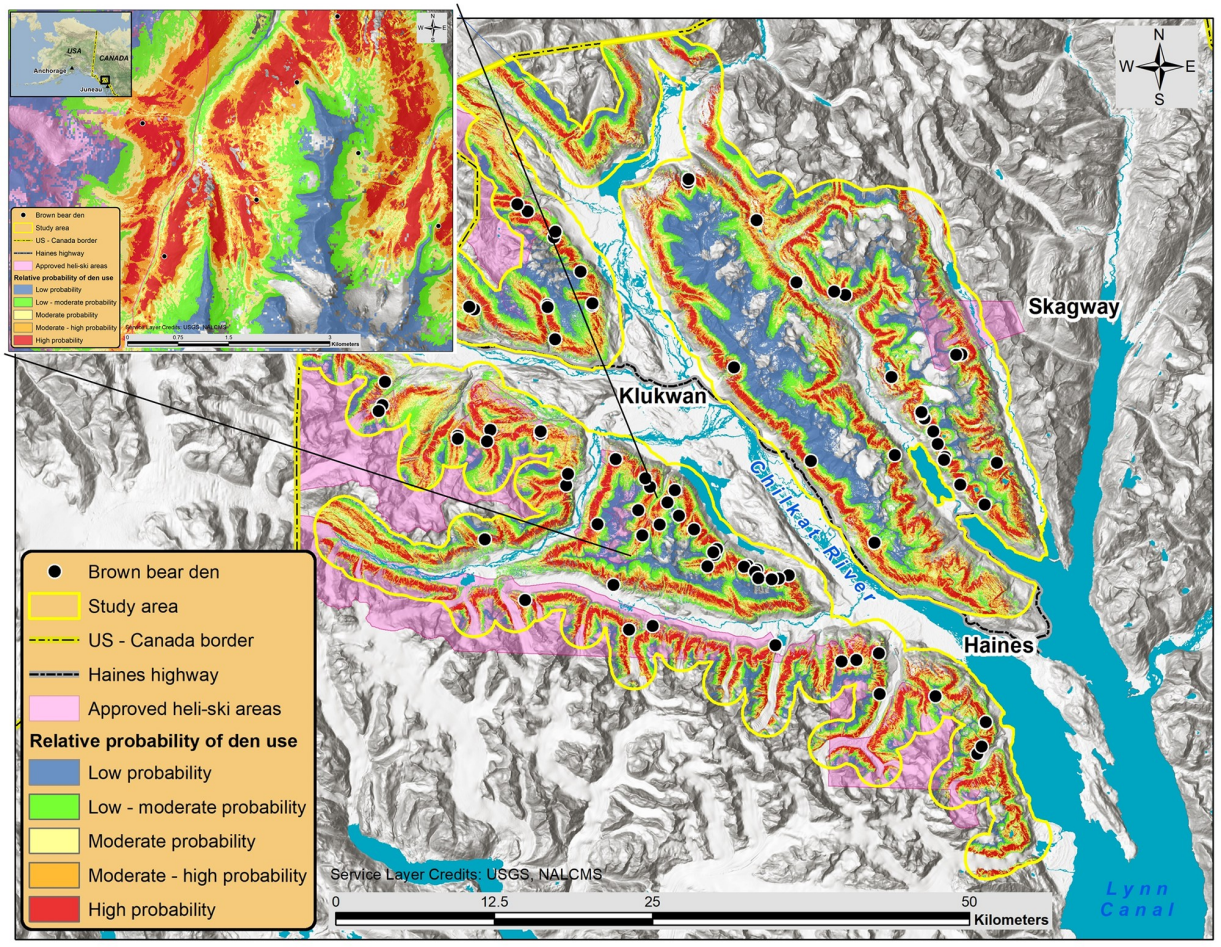
Brown bear resource selection function model (RSF) based on terrain and climate factors important to brown bears denning in alpine and subalpine habitats near Haines, Alaska, U.S.A., 2008–2017. Generalized bear den locations are depicted by black circles, the study area is outlined by a solid yellow line, approved heli-skiing areas are shaded violet, and relative probability of den use is color coded. The map inset displays a zoomed in portion of the study area to detail the high-resolution detail of the RSF surface. Land cover information is republished from [57] under a CC BY license, with permission from Commission for Environmental Cooperation, original copyright 2020.

**Fig 4:**
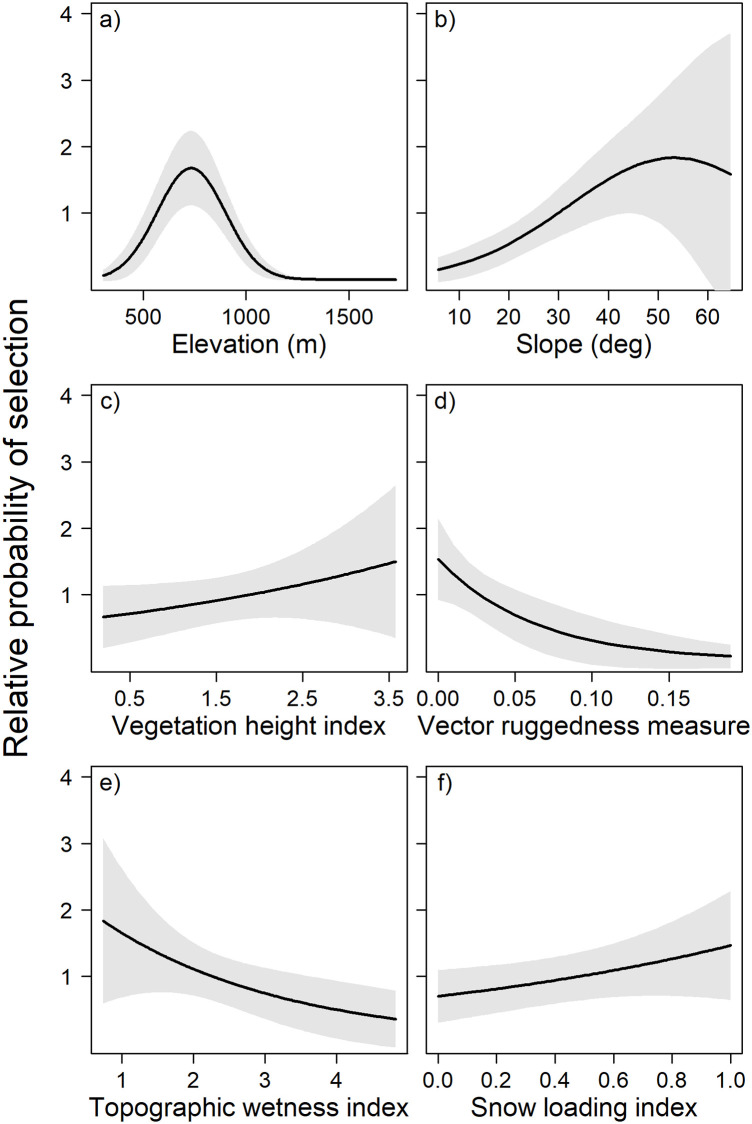
Average effect of individual continuous factors from resource selection function model of brown bear alpine den site selection near Haines, Alaska, U.S.A., 2008–2017. Black lines represent the relative probability of den site selection and gray shading signifies 95% confidence intervals.

Brown bear dens were broadly distributed throughout the study area, and enabled generation of an RSF surface capable of spatially delineating relative probability of den site selection at a relatively high degree of resolution (Fig 3). The RSF surface contained 421.2 km^2^ of prime denning habitat (moderate-high to high probability use), accounted for 40% of the study area, and included 85% of the den sites. An RSF score for each den site indicated that 56 dens (63%) were located in the high relative probability of use category, 20 dens (22%) in moderate-high, nine dens (10%) in moderate, four dens (4%) in low-moderate, and zero dens were located in the low category. Bears were selective in their use of elevation and slope for denning, and inclusion of their quadratic forms in the top model indicate that bear den sites were not situated near the extremes of these terrain factors. Dens were excavated in the lower elevational extent of available alpine terrain (Fig 4a). Brown bears positively selected moderately steep slopes and the individual factor effect graph indicated a peak in the slope RSF score at 53.3° (Fig 4b). Bears also favored den sites in more smooth, less rugged terrain with better drained soils, as specified by the negative relationship between the VRM and TWI factors and RSF scores (Fig 4d and 4e).

### Heli-skiing—Denning habitat overlap

Despite the presence of prime denning habitat, we observed fewer dens than expected within the approved heli-skiing areas and found in-bounds dens only near the perimeter of those areas. We surveyed nearly half of the approved ski area (437 km^2^) and determined that 28% (117.7/421.2 km^2^) of all available prime brown bear denning habitat was overlapped by permitted heli-skiing areas. We detected 66 dens (74%) outside of the heli-skiing areas and observed 23 dens (26%) within the boundaries, 91% of which were located in prime denning habitat. Two-thirds of the dens located in the heli-skiing areas were within 1 km of the ski area boundary (Fig 3).

**Fig 5:**
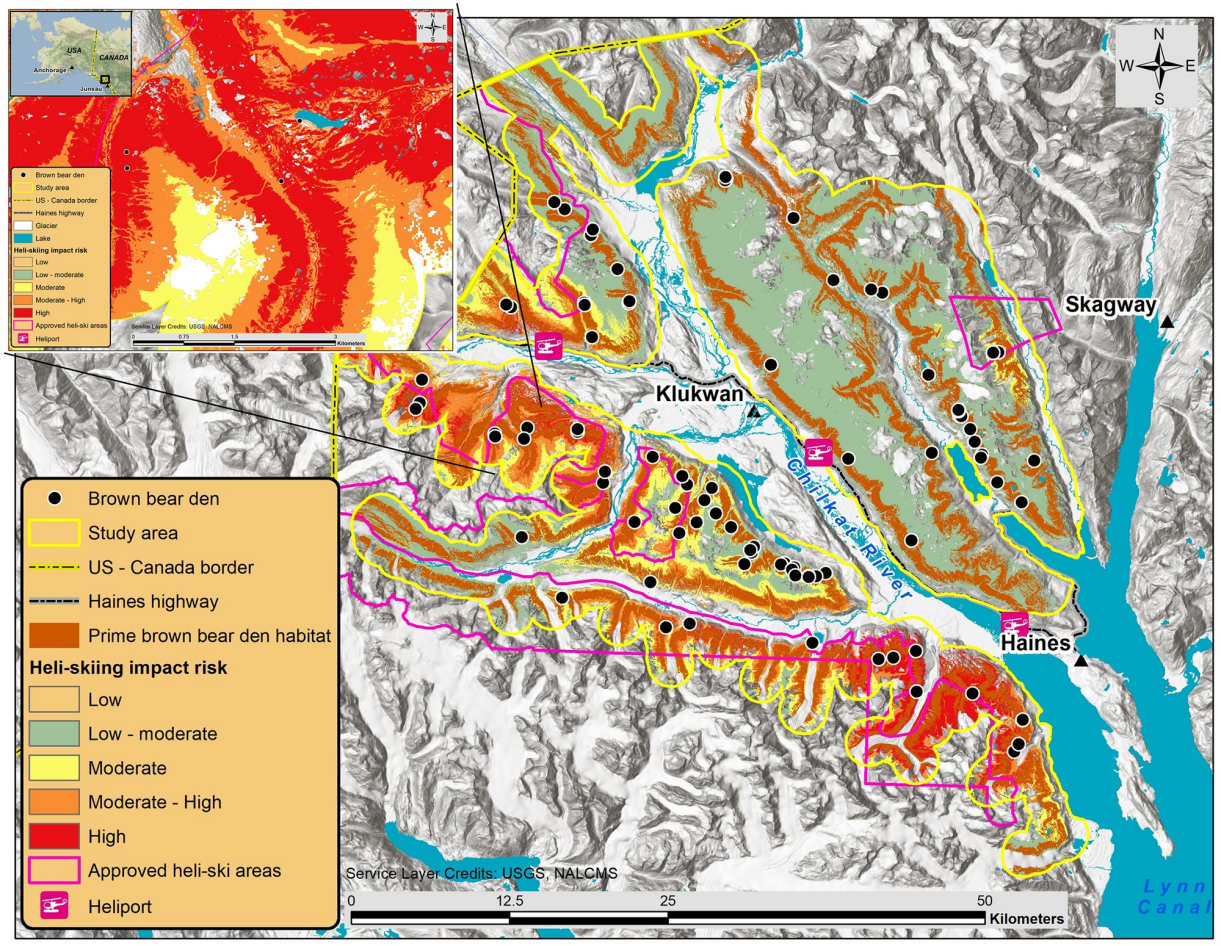
Heli-skiing impact risk to prime brown bear denning habitat near Haines, Alaska, U.S.A., 2008–2017. Areas where prime denning habitat was overlapped by high helicopter intensity are colored red, other areas are color coded to show the potential risk to denning habitat from heli-skiing. Generalized brown bear den locations are denoted by black circles, study area is delineated by the solid yellow line, approved heli-skiing areas are outlined in violet, and authorized heliports are identified. Land cover information is republished from [57] under a CC BY license, with permission from Commission for Environmental Cooperation, original copyright 2020.

The heli-skiing utilization distribution showed helicopter activity concentrated in three ski areas proximal to heliports. There was considerable overlap between helicopter activity and brown bear den habitat (S1 Table). We located more than half of the dens in areas characterized by low intensity helicopter activity, while only four dens were observed in habitat subjected to the highest helicopter intensity. The heli-skiing impact risk surface showed the potential risk to denning bears increased with higher intensity helicopter use, proportional to the resource selection value of the denning habitat. We found 35 (39%) dens within the two highest heli-skiing impact risk categories (Fig 5).

## Discussion

Brown bears selected a specific suite of terrain and climate factors that contributed to optimal maximization of thermal efficiency. Alpine habitat occurs across a wide range of elevations and slopes, yet brown bears occupied den sites within distinct ranges and combinations of slope and elevation. Concordant with other studies of den site selection, bears occupied moderate alpine elevations to ensure deep and stable snow coverage [94], selecting dens sites that were located high enough to maintain cold temperatures for snow accumulation and thermal insulation but avoided extreme elevations where terrain may become windswept and contain substrate less suitable for excavation (i.e., bedrock or glacier) [34, 48, 52, 66, 95]. In this study bears denned at a mean elevation (755 m) similar to collared brown bears studied on nearby Admiralty Island (713 m) [17] and Kenai Peninsula (646 m) [34], suggesting that preferred thermal security and snow condition requirements for denning are found at similar elevations in our coastal mainland Alaska site. Bears excavated dens on slopes that allowed them to dig nearly horizontal entrances, which can help stability and conserve heat loss by trapping warm air in the nest cavity [45, 48]. Dens must be thermally efficient, and we found that dens were commonly located on well-drained terrain in aspects offset from prevailing winter winds. Bears selected dens in these landscapes which were conducive to snow loading as thermal insulation protects from direct exposure to harsh winter weather conditions [27, 96]. Given the complex mechanisms associated with the redistribution of snow by wind and topography [95, 97], and the decrease in atmospheric moisture and snow deposition at higher elevations [94], we suggest developing a more empirical snow model in future den site selection modeling efforts [98, 99].

Identifying dens via aerial surveys and measuring their landscape attributes with remotely sensed data can be a cost-effective method for predicting brown bear den selection patterns in expansive mountain landscapes [49]. Given the need for science-based information to help inform heli-skiing management decisions, we demonstrate a fiscally- and time-efficient approach to identifying alpine and subalpine brown bear denning habitat. It is important to ensure surveys follow a consistent protocol, coincide with den emergence, and are conducted during favorable snow conditions. Such an approach helps confirm that detection probabilities are consistent within open, alpine habitat survey frames, and robust to detection bias issues. Since our primary goal was to quantify relative probability of selection of den site covariates, incomplete detection does not represent an important consideration, as compared to other aerial survey applications involving population estimation (i.e., density estimation via distance sampling) [100]. We followed a standardized protocol to maintain constant observer bias and treated each aerial survey as a relative indirect count, rather than an absolute estimate of den abundance. We expect a number of bear dens were not detected, because bears had either not yet emerged from their den or occupied habitat that inhibited detection. Adult female bears with offspring are typically the last cohort to emerge from their winter den and potentially were underrepresented by the dens we observed. However, regional data on timing of den emergence indicate that most bears denning at high elevation were likely available for sampling [17, 101]. We also acknowledge geographical variation in bear den selection strategies and recognize that our model does not represent bears selecting den sites at lower elevations (which is beyond the scope of our specific management interests). Recent GPS-collar studies in SEAK documented a substantial proportion of den sites at low elevation, illustrating the under-valued contribution of coastal forested habitats to denning [101, 102]. Additional GPS den locations would improve factor coefficient precision, enable den habitat prediction at lower elevations, and could address concerns with detection bias. While there is imperfect den detection, we believe the den characteristics observed across multiple years under varying survey conditions provide a representative sample of brown bear alpine den habitats selected in this region.

Habitat selection models are sensitive to the scale of habitat factors [103] and we analyzed den site selection using data measured at two spatial resolutions, including a land cover classification (30-m) and fine-scaled terrain factors derived from IfSAR data (5-m). We predicted that bears would select for land cover type that promoted den structure (i.e., shrub) as vegetation roots would enhance the structural stability of the soil and minimize den collapse [48]. However, models including land cover covariates performed poorly in this heterogenous landscape and were excluded from final model selection. It is possible that the scale at which dens were selected was finer than the accuracy and resolution of the available land cover classification. This finding is similar to past habitat selection studies demonstrating the negative influence of lower resolution data layers on model performance [103, 104]. Our utilization of fine-scaled vegetation height data (i.e., VHI) improved our ability to predict den habitat and served as a good surrogate for the vegetated habitats that potentially improved the structural stability of the den sites.

Understanding how climate change and snow conditions affect the timing of den entrance and emergence has implications for managing winter recreation [105]. Diminishing snowfall and earlier onset of warmer spring temperatures have resulted in earlier den emergence and shorter denning duration [50, 106]. For example, during a long-term study of bear denning ecology, as maximum average temperature increased 4°C, bear den emergence shifted 10 days earlier [106]. In Southeast Alaska, adult males typically den for the shortest duration, entering the den later (Nov-Dec) and exiting earlier (late March-April) [17]. Adult females with cubs den the longest and typically enter the den in mid-Nov and exit the den between mid-April and early-May [17]. Annual variation in den entrance and emergence dates is based on food availability in the autumn [53, 96], temperature, spring snow depth [69, 107], and disturbance [21]. The effects of heli-skiing disturbance during early season and late season hibernation may be pronounced and have the potential to cause den abandonment, resulting in energetic costs which may lead to weight loss and decreased cub survival [18, 21, 27]. Reynolds et al. [27] documented five cases where helicopter activity in the early denning season caused den abandonment, and Swenson et al. [18] found that dens abandoned in the early winter led to cub mortality and travel up to 30 km to establish a new den site. Early den emergence in the spring has been documented to negatively affect cub survival with a greater likelihood for female bears to successfully produce and raise cubs when denning duration lasted as few as 15 days longer than females without cubs [108]. In this study, we documented evidence of late season den abandonment due to disturbance from heli-skiing within 400–1,000 m from the den site and subsequent avoidance of previously favored den habitat. The animal realized short-term energetic costs associated with early den emergence, and potentially long-term consequences from future avoidance of favorable den habitat that became authorized for heli-skiing.

In SEAK, the cumulative impacts of heli-skiing, industrial mine development, and timber harvest may contribute to increased risk of disturbance and jeopardize brown bear population productivity [109]. Schoen et al. [17] found reproductive females selected dens further from helicopter activity associated with mining and gradually increased denning distance from developed mine activity. They cautioned that helicopter traffic during periods of den entry and emergence should be routed away from denning areas. Where helicopter activities must overlap high use bear denning habitats, best practices recommend limiting flight duration and frequency and maintaining altitude > 500 m above ground level [30]. Mitigation measures on the North Slope oilfields in Alaska require operators to maintain a 0.8 km buffer from known brown bear dens during exploration, production, and other mobile activities and 1.6 km from polar bear dens [110]. Logging activities in winter have also resulted in bear disturbance, coinciding with a greater probability of offspring mortality [18]. Where roads and industrial activities are in close proximity to denning habitat, bears select dens 1–2 km beyond those disturbances [21, 34, 66]. As development and disturbance continue to increase, managers can mitigate the effects on bears by increasing the distance between disturbance activities and denning habitat.

As the number of winter recreationalists accessing the alpine continues to grow, the model we developed may offer a way to reduce human-bear conflicts, especially the potential for surprise encounters near den sites that often result in human injury [111]. In 2016, warm temperatures and minimal snowpack rerouted a University of Alaska mountaineering class from their normal field site to our study area. A surprise encounter between the professor and a brown bear near its den site resulted in the instructor being severely mauled. The incident occurred in terrain that our model predicted to be prime denning habitat. In the future, we envision our model can also be used to further benefit the public by identifying appropriate places for winter backcountry activities.

Our analyses provide evidence of apparent avoidance of favorable denning habitat in areas with frequent heli-skiing disturbance as we observed fewer dens than expected within the approved heli-skiing areas. To put the potential heli-skiing impact into population perspective we estimated the number of dens that could theoretically be located in alpine and subalpine habitat within this game management unit. Using an estimated population of 400 bears [63], and considering reasonable assumptions on population demographics, with an anticipated 40% of dens situated above the forest line, we would expect 128 alpine dens across the game management unit (S2 Table). Portions of the study area have experienced heli-skiing activity for two decades and the possibility exists that in some drainages bears have been displaced from prime den habitat. Exclusion from prime denning habitat, and subsequent use of suboptimal denning sites, could affect natural distribution patterns and lead to population level declines in reproduction and survival. Alternatively, it is possible that bears select dens in suitable habitat in the autumn without considering previous denning experience, or that we observed dens after bears relocated away from heli-skiing disturbance. Displaced bears could den elsewhere in the alpine or at lower elevations in forested habitat, but the availability of suitable and unoccupied dens in either setting is unknown. The degree to which den site selection is a learned behavior and the ability of individual bears to select suitable dens in different settings is also unknown, yet data from collared brown bears indicate that den sites can be re-used for multiple years [96].

### Management implications

We provide results and a set of methods that can inform management actions when optimizing trade-offs associated with heli-skiing regulation and brown bear conservation. As denning habitat may be influenced disproportionately by the intensity of heli-skiing activity, minimizing disturbance to bears requires reasonable measures to avoid prime denning habitats. Given the potential for mechanized winter recreation to affect survival and productivity of brown bears and other wildlife, resource managers should incorporate information on disturbance effects into permitting decisions. We propose the following recommendations be considered when operating within brown bear denning areas in coastal Alaska and Canada. Considering the scientific evidence presented here and the intensity of heli-skiing disturbance, a prudent guideline used to mitigate disturbance to other wildlife species, would be to establish a set-back distance of 1.5 km between prime denning habitat and heli-skiing activity [112–114]. While not all disturbance can be avoided, we recommend the heli-skiing industry commit to establishing best management practice standards for helicopter operation with prescribed flight corridors and predictable approach vectors through regions with sensitive wildlife habitats. Establishing minimum helicopter flight altitudes and separation distances from prime bear denning habitats could mitigate the effects of aircraft disturbance on denning brown bears. Designating control areas where heli-skiing activities are restricted would allow for comparative studies on the direct effects of disturbance. Monitoring the spatial intensity of heli-skiing activity with respect to bear denning habitat would vastly improve managers capability to understand disturbance effects and bear population dynamics. As managers balance the needs of the economy, community, and brown bear conservation, our findings provide answers to questions about where important habitats occur on the landscape. This empirical framework helps advance our understanding of alpine brown bear denning habitat and these resources will inform decision makers developing winter recreation land use policies.

## Supporting information

S1 Fig Comparison of brown bear den elevation between distinct geographical mountain ranges in Haines, Alaska, U.S.A. 2008–2017. (TIF)

S2 Fig Polar chart of den aspect for 89 brown bear den sites located by aerial survey and GPS collars in Haines, Alaska, U.S.A., 2008–2017. (TIF)

S1 Table Brown bear denning habitat summarized by the areal extent of overlap with the intensity of heli-skiing activity in the 1,052 km^2^ study area in Haines, Alaska, U.S.A. (TIF)

S2 Table Estimated annual number of brown bear dens in alpine habitat in Game Management Unit 1D in Haines, Alaska, U.S.A., based on estimates from Miller [63]. (TIF)

S1 File (PDF)
