# Supplementary figures and images for: Steep and deep: Terrain and climate factors explain brown bear (Ursus arctos) alpine den site selection to guide heli-skiing management
Source: PLoS One. 2020 Sep 23;15(9):e0238711. doi: 10.1371/journal.pone.0238711 (PMC7511016; doi:10.1371/journal.pone.0238711)

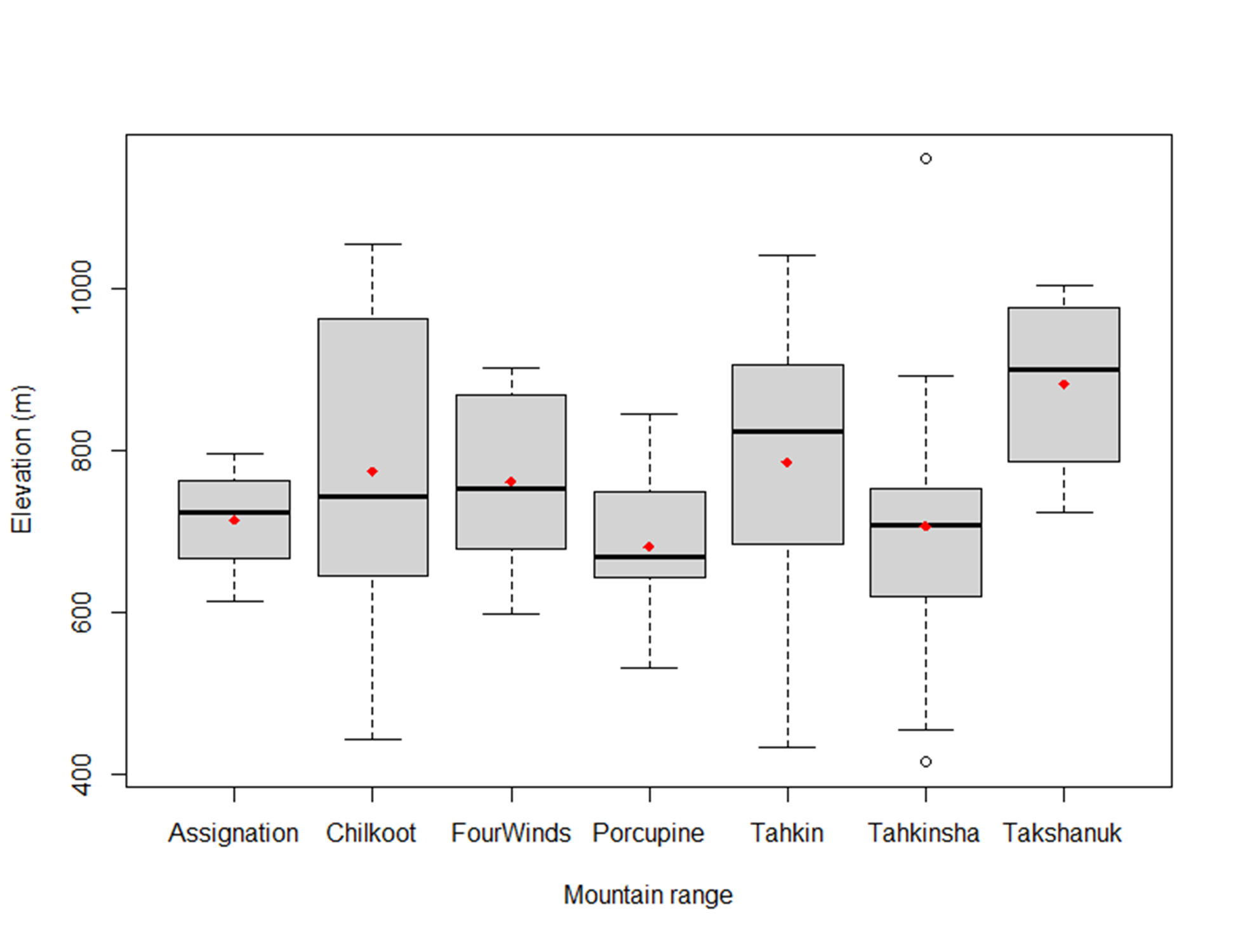

Supplement: S1 Fig — (TIF) [file pone.0238711.s001.tif]

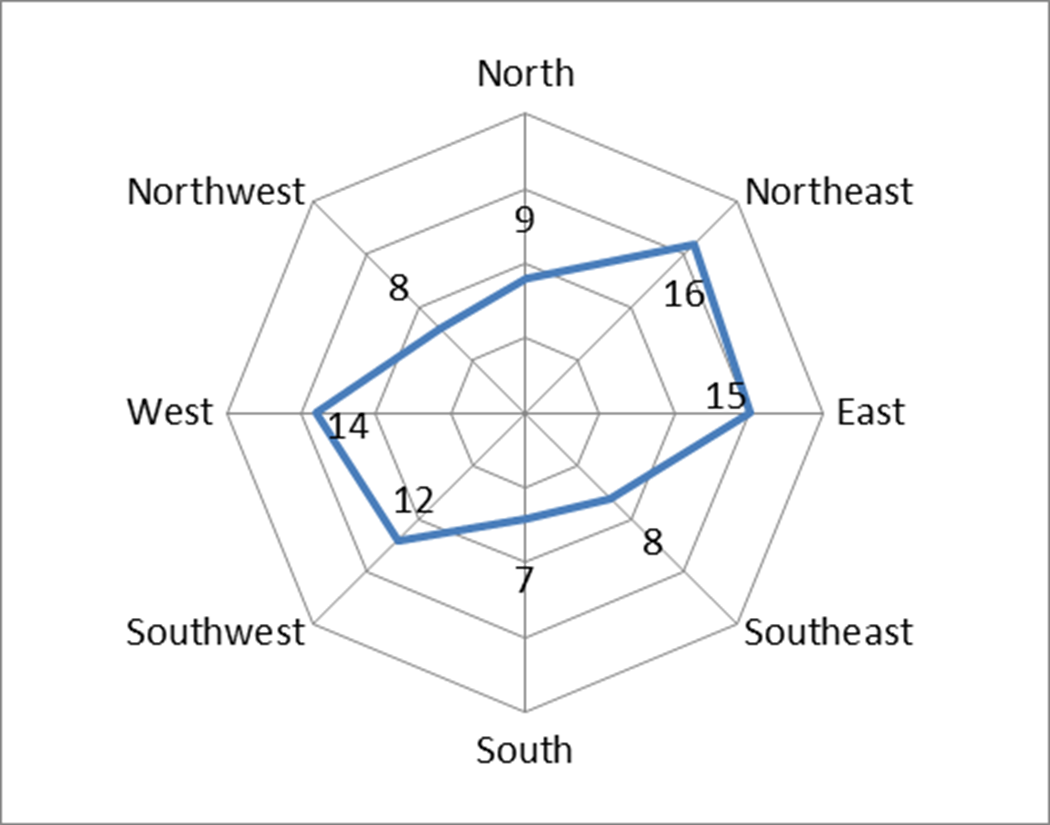

Supplement: S2 Fig — (TIF) [file pone.0238711.s002.tif]

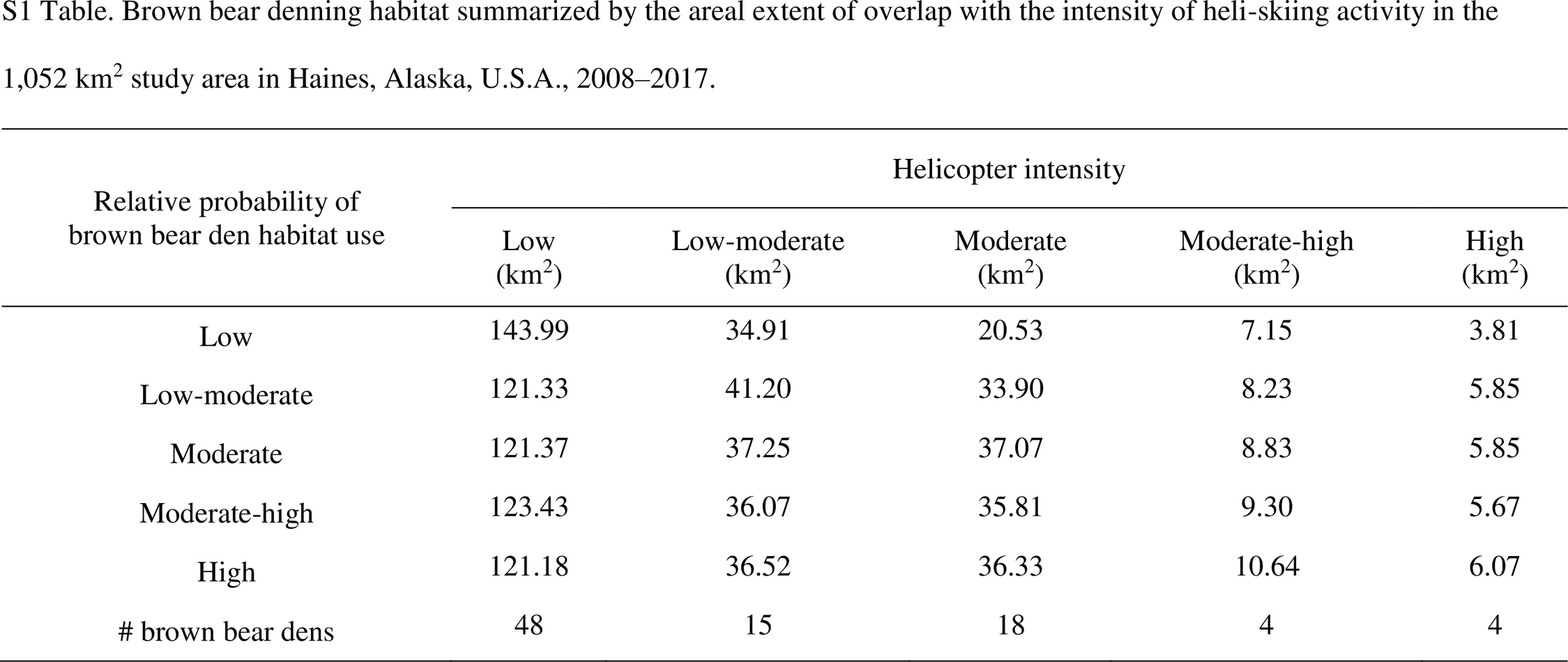

Supplement: S1 Table — (TIF) [file pone.0238711.s003.tif]

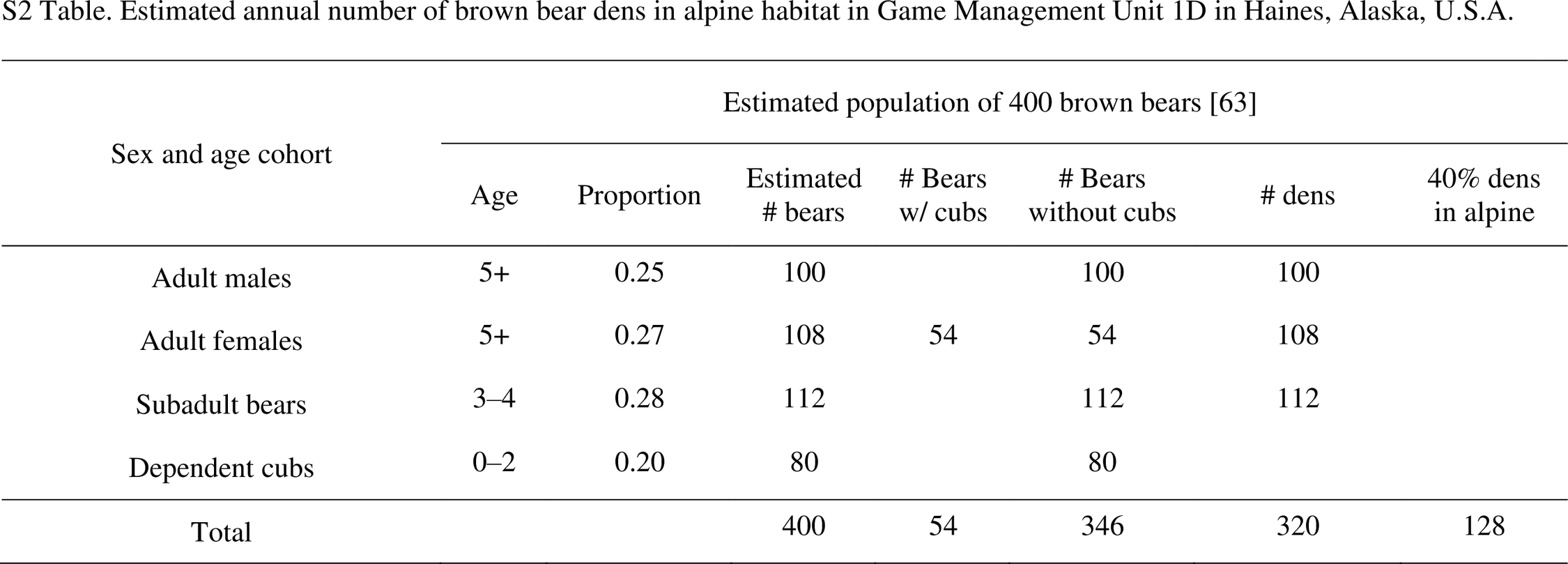

Supplement: S2 Table — (TIF) [file pone.0238711.s004.tif]
