# Supplementary material for: Steep and deep: Terrain and climate factors explain brown bear (Ursus arctos) alpine den site selection to guide heli-skiing management
Source: PLoS One. 2020 Sep 23;15(9):e0238711. doi: 10.1371/journal.pone.0238711 (PMC7511016; doi:10.1371/journal.pone.0238711)
Supplement: S1 File — (PDF) [file pone.0238711.s005.pdf]

Request for Permission to Publish Content under CC-BY License

Dear Rights Holder or Representative,

I have submitted a paper for publication in a PLOS journal, and wish to include the content listed below in the paper. I'm hereby requesting your (or your company's or institution's) permission to include the content in my paper. Please note that all PLOS journals are published under a Creative Commons Attribution License (CC BY), which allows for unrestricted use and distribution, even commercial, as long as attribution is given to the creator or rights holder of the content. See https://creativecommons.org/licenses/by/4.0/.

To grant me permission to use the content in my PLOS paper, please fill in the information below and then scan the completed form and send it to me at my email address.

Thank you.

My name:

Orlando Cabrera-Rivera

My email address:

ocabrera@cec.org

Description of the content which I'm seeking permission to use (citation and/or title, and pasted screen shot, if applicable):

Commission for Environmental Cooperation. "2015 Land Cover of North America at 30 Meters", North American Land Change Monitoring System, Ed. 1.0. 2020.

Link to the Content:

cec.org/tools-and-resources/map-files/land-cover-30m-2015-landsat-and-rapideye

* * *

On behalf of myself or the rights holder, I hereby grant the permission sought herein.

Signature of Party Granting Permission:

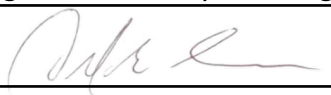

Date:

11 May 2020

Printed Name and Title:

Orlando Cabrera-Rivera, Head of Unit, Enviromental Quality, Commission for Environmental Cooperation
